# Why Use Ultrashort Pulses in Ophthalmology and Which Factors Affect Cut Quality

**DOI:** 10.3390/medicina57070700

**Published:** 2021-07-08

**Authors:** Bojan Pajic, Brigitte Pajic-Eggspuehler, Christian Rathjen, Mirko Resan, Zeljka Cvejic

**Affiliations:** 1 Eye Clinic ORASIS, Swiss Eye Research Foundation, 5734 Reinach AG, Switzerland; brigitte.pajic@orasis.ch; 2 Department of Physics, Faculty of Sciences, University of Novi Sad, Trg Dositeja Obradovica 4, 21000 Novi Sad, Serbia; zeljka.cvejic@df.uns.ac.rs; 3 Division of Ophthalmology, Department of Clinical Neurosciences, Geneva University Hospitals, 1205 Geneva, Switzerland; 4 Faculty of Medicine, University of Geneva, 1205 Geneva, Switzerland; 5 Faculty of Medicine of the Military Medical Academy, University of Defense, 11000 Belgrade, Serbia; resan.mirko@gmail.com; 6 Ziemer Ophthalmic Systems, 2562 Port, Switzerland; christian.rathjen@ziemergroup.com

**Keywords:** femtosecond laser, ultra-short pulses, cut quality

## Abstract

The power density of femtosecond lasers and exposure time to the tissue are crucial for a successful procedure in terms of safety and precision. The reduction of the pulse duration allows reducing the quantity of the energy to be delivered to the tissue for disruption with strongly diminished mechanical and thermal collateral damage. The cutting effect of ultra-short pulses is very precise, minimally traumatic, safe, and predictable. Future developments will lead to further energy reductions to achieve optical breakdowns. However, the pulse length cannot be shortened arbitrarily because below 100 fs nonlinear effects can change the process in an unfavorable way. Compared to manual-conventional cataract surgery, femtosecond laser-assisted cataract surgery (FLACS) shows many advantages in clinical application, especially with regard to precision and tissue protection. The femtosecond laser has become particularly important and has made the overall procedure safer when we deal with complex cataract cases such as subluxated lenses. We provide an overview of the evolution of femtosecond laser technology for use in refractive and cataract surgeries. This article describes the advantages of available laser platforms with ultrashort pulses and mainly focuses on the technical and physical backgrounds of ophthalmic surgery technologies.

## 1. Introduction

The age of small-cut technique in cataract surgery was heralded by the introduction of the phacoemulsification surgical technique in 1967 [1]. In the early of the 1970s, active thinking about laser cataract surgery had already begun. The only available lasers caused a thermal effect and were mainly used to treat retinopathy. With the advent of Q-switched lasers, pulse duration reduced, which significantly decreased associated thermal effects. This led to a massive shortening of the laser pulse duration to a level to nanoseconds or picoseconds. Q-switched ruby and neodymium lasers used at that time the energy of a single pulse up to 0.3 J with a pulse length of 20 nanoseconds. Since 1972, the Q-switched laser-phakopuncture has been used on the human eye. In all eyes, the surgery had to be continued and ended conventionally and the eye did not suffer any damage from the laser. It was also found that the lens was resorbed in soft cataracts after the laser-phakopuncture. However, this required several sessions. In present time, with this type of cataract laser-phakopuncture, it is possible to obtain an aphakia without conventional surgery [2].

The Nd:YAG laser was introduced for capsulotomy at the beginning of the 1980s. Noteworthy was intraocular cutting without the bulb opening at all. A small diameter of the laser beam allowed a very precise application, that the risk of damage in the eye, or the intraocular lens was minimized [3]. It was also found that collateral damage depended directly on the laser pulse energy [4,5]. For further reduction of the pulse energy, it was necessary to shorten the pulse duration from nanoseconds to picoseconds [6,7,8]. In an in vitro experiment, it was shown that reducing the pulse duration from 6 ns to 30 ps reduced the threshold for plasma formation by a factor of 13 to a level of 15 μJ [6]. This step toward a pulse duration of picoseconds and a microjoule energy level has opened up new potential indications for use of Nd:YAG laser in cataract surgery or vitreoretinal operations [9].

The first commercially offered laser for cataract surgery was an erbium (Er):YAG. With this kind of laser the small-incision technique in cataract surgery was further reduced and became less invasive. In a laboratory study, the interaction of 2940 nm wavelength of Er:YAG and the 2790 nm wavelength of Er-YSGG were examined in human crystallin lens tissue [10]. As human lens tissue consists of 63% water [11], and due the fact that the water has strong absorption on 2950 nm [10], the idea was to photoevaporrate the lens using mid-infrared laser radiation. Both lasers, Er:YAG (2940 nm) and Er:YSGG (2790 nm), have a comparable emission wavelength, while the water absorption coefficients for both lasers are 13,000 cm^−1^ and 7000 cm^−1^, respectively [12]. The photoevaporation threshold for the Er:YAG laser is 1.4 J/cm^2^ and 5.5 J/cm^2^ for Er:YSGG soEr:YAG lasers have a significantly lower energy density needed for the photoevaporation (4-fold), than the Er:YSGG [10]. As a result, the damage zone in the tissue is smaller for Er:YAG lasers (range: 4–9 μm) as compared to the Er:YSGG laser (range: 10–22 μm).

The potential advantages of erbium lasers in lens-removing cataract surgery include their safety, a relatively fast ablation regardless of the cataract density, and especially their small size, which allows a small-section technique [10]. The introduction of the pulsed Q-switch of the laser medium at both laser sources could significantly reduce the damage zone in corneal tissue [12]. Furthermore, the water more intensively absorbed the irradiation of the Er:YAG laser than laser irradiation of the Er:YSGG [10]. Therefore, the Er:YAG laser source has proved to be more suitable for the intended purpose overall.

The Er:YAG laser still had to show superiority over conventional phacoemulsification in cataract surgery. In a pilot study, three capsule ruptures occurred in 40 cataract surgeries [13,14]. These happened due to dynamics of the laser–tissue interaction process of the Er:YAG laser and the learning curve of the surgeon. The laser beam penetration of an Er:YAG laser is calculated at a depth of 1 μm. However, the ablation process is dynamic, so the penetration is much higher than the calculated 1 μm [15]. The absorption of the laser energy in the first micron leads to explosive evaporation and the formation of a cavitation bubble [16]. Microjet formation, which traverses the first cavitation bubble, forms a second bubble. Energy propagation penetrates more than 1 mm and ruptures the lens capsule [17]. The boundary of the potentially destructive wave of propagation energy was not visible to the surgeon, so the procedure led to a blind flight. Various manufacturers sold Er:YAG lasers for many years; however, this technology was not successful and was no longer developed.

At the end of the 1980s, a neodymium-doped yttrium aluminum garnet (Nd:YAG) laser with a wavelength of 1064 nm emerged as an alternative to Er:YAG, as an indirect system for cataract surgery [18,19,20]. The pulsed Nd:YAG laser strikes a titanium target, which is integrated into the aspiration and irrigation probe and generates controlled shock waves. This results in the lysis of the cataract nucleus, which is consecutively aspirated by the system [20]. With this system, the laser source is not directly exposed to the surrounding tissue, which could potentially be considered gentle. While cataract surgery could be carried out with Nd:YAG, the lack of applicable laser energy limited its widespread use.

The first experimental applications of femtosecond laser technology were in the late 1980s, when the femtosecond laser was studied on the retinal tissue of animals [21]. The in vitro application of the femtosecond laser to human corneal tissue was first described in 1994 [22]. The femtosecond laser can only perform its cutting function in transparent tissue. For this reason, its target tissue or target area is the cornea and lens. The cornea is the anterior transparent part of the eye capsule; its very specialized tissue is of great importance for vision due to its avascularity. The lens has a biconvex shape. It has no vascular system, and its refractive power is variable due to its deformability. It is transparent in this sense. It should be mentioned, however, that the femtosecond laser can also cut through corneal scars, for example, if they are not too dense. This is also true for cataract, that segmentation of a lens opacity is very well possible except for very dense lens nuclei.

Nine years later, in 2003, the first commercial femtosecond laser was introduced to the market, which opened a new chapter in corneal and cataract surgery. Initially, the femtosecond laser was used in refractive and corneal surgery, which significantly increased surgical precision and safety compared to a microkeratome [23,24]. In 2008, its application was expanded to also cover the cataract surgery [25]. Its application has brought better predictability, safety, and refractive outcome [26].

In recent years, many studies have been conducted on the femtosecond laser technology. In the field of refractive surgery, the advantages of femtosecond technology compared to microkeratome LASIK are well documented [27,28,29,30,31,32,33,34]. The femtosecond laser provides higher precision, better centering of capsulotomy, lower effective phaco time (EPT), and clear corneal incision [35,36,37,38,39,40,41,42] in cataract surgery. With the ultrashort pulses—10^−9^ to 10^−12^ s or less—the femtosecond laser possesses great potential not only in terms of higher precision and safety of treatment, but also in terms of completely new treatment methods and principles.

## 2. Materials and Methods

For this review article, a comprehensive literature search of Web of Science (all years), PubMed, the metaRegister of Controlled Trials (www.controlled-trials.com, accessed on 1 December 2020), ClinicalTrials.gov (www.clinicaltrial.gov, accessed on 1 December 2020), and World Health Organization International Clinical Trials Registry Platform (www.who.int/ictrp/search/en, accessed on 1 December 2020) was conducted using keywords: ultrashort pulses in ophthalmology, laser cataract surgery, femtosecond laser cataract surgery, optical breakdown, photodisruption, cut quality. We selected only English language papers relevant to this review. In addition to this, some of the authors of this article have extensive surgery experience in everyday practice with femtosecond laser. 

## 3. Ultrashort Pulsed Femtosecond Laser: Mechanism of Laser–Tissue Interaction

### 3.1. Photodisruption

Photodisruption occurs when a threshold of optical breakdown exceeds $10^{11}$ W/cm^2^ at which a laser-induced optical breakdown occurs [43]. It is the principal mechanism in the application of ultrashort pulsed femtosecond lasers in ophthalmology.

**Figure 1:**
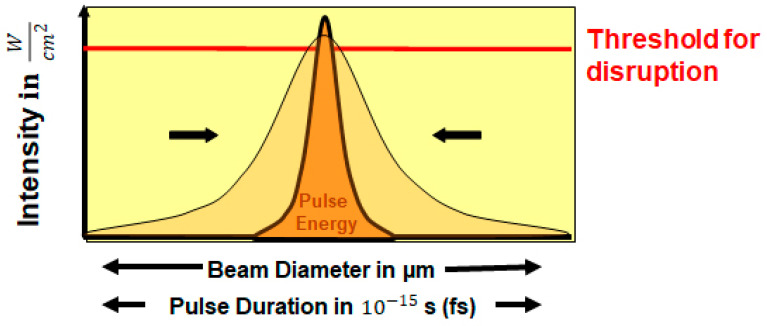
By shortening the pulse duration, the pulse energy to reach threshold intensity is reduced and thus the potential side effects are also reduced. The intensity is defined with W ($\frac{J}{s}$) as the power and the applied area in ${\mathrm{cm}}^{2}$.

Laser beams with high-photon density and very short pulse duration lead to nonlinear absorption. Because of the multiphoton ionization and cascade ionization, the absorbed energy becomes sufficient to exceed the threshold for optical breakdown. The rate at which the multiphoton ionization occurs is proportional to the laser intensity in the focus and the number of photons required to ionize the tissue molecules [44]. Free electrons in the material, on the other hand, absorb a lot of photon energy in a non-resonant process, and the electrons are consequently accelerated. This process is called inverse bremsstrahlung (i.e., “breaking radiation”) and takes place in the presence of a third particle. After repeated absorption, even the accelerated electrons gain increasingly more kinetic energy, such that they can ionize other molecules and even create free electrons in much larger numbers or concentration. If the intensity of laser light is enough to overcome the loss of the electrons through diffusion or recombination, then an avalanche effect arises. Consequently, there is a sharp increase in electron density for a short period of time. When free electrons reach a critical value of approximately $10^{18}$–$10^{20}$/cm^3^, a cloud of ions and free electrons is formed in the laser beam focus, called plasma. In this case, an optical breakdown is generated. The threshold exposure requires less pulse energy for forming an optical breakdown when the pulse duration is shortened (Figure 1).

For pulse duration below 200 fs, the threshold exposure remains approximately constant. Because of the sudden heating of the plasma and the subsequent recombination of the ions and electrons in the tissue, a shock wave of 10 MPa is emitted from the focus volume, which quickly loses energy [43]. Because the plasma lifetime is short—a few picoseconds only—no thermal energy is released to the surrounding tissue. After recombination of the plasma, a remaining gas bubble expands due to its internal gas pressure. The so-called cavitation bubble is formed at the focus of the laser beam and consists essentially of ${\mathrm{CO}}_{2},{\;N}_{2},{\;H}_{2}O$. Due to internal and external mechanical forces that are not in equilibrium, its size expands, oscillates, and finally collapses to a small gas bubble The maximum size of the cavitation bubble correlates with the energy of the laser pulse and can be significantly larger than the focus volume itself if large focus diameters and long pulse durations are applied. Higher pulse energy results in large cavitation bubbles and a strong tissue-disrupting effect. After the cavitation bubble has collapsed, a small bubble of gas remains at the focus volume, which is defined by the Rayleigh range and beam waist of the laser beam. The gas is finally resorbed by the surrounding tissue [45].

### 3.2. Cutting the Tissue

Cutting the tissue is achieved by placing individual laser shots next to or overlapping to each other, thus creating a continuous cut in the tissue. The greatest possible precision is achieved with minimal cavitation bubble size, and therefore small pulse energies. As the triggering of a laser-induced optical breakdown depends on the material (threshold for photodisruption is given in W/cm^2^) only smaller beam diameters and shorter pulse duration can be envisaged to lower the pulse energy (given in Joule) according to Figure 1. Conversely, if the tissue area separated per laser pulse decreases, a higher number of pulses is needed to achieve the same incision size. Without increasing the repetition rate of the laser system, this would lead to an increase in the duration of treatment. Therefore, high repetition rates must be applied when small pulse energies are used. The quality of the cut also depends on the spatial distance between the laser pulses as a function of their energy [6]. Cavitation bubbles can have a diameter of up to one millimeter when high pulse energies and nano second (ns) pulses are applied. Theoretically applying high pulse energies at large spatial distances would lead to a very low number of applied doses (in J/cm^2^). However, such an energy-optimizing strategy would lead to a clinically unacceptable precision and cut quality. Luckily, besides a better precision, side effects of femtosecond lasers have turned out to be minor for systems with lower pulse energies and high repetition rates [46]. For this reason, the ultimate goal must be to keep the pulse energy low and not reduce the total amount of applied doses, which is an inappropriate parameter for cut quality.

When the threshold intensity is achieved and the breakdown process has started, attempts to further increase the intensity are of no benefit, as they would ultimately lead to unnecessarily large cavitation bubbles.

If both the duration and area of the irradiation are limited, i.e., a short laser pulse duration with a strong focus is applied, the intensity threshold is reached with much less energy (Figure 1). This is the basic idea behind ultrashort pulses: as soon as the nonlinear process is initiated, it should also be terminated as quickly as possible, because otherwise the energy used will lead to side effects such as large cavitation gas bubbles, shock waves, and high temperatures.

## 4. Factors Influencing the Cut Quality

### 4.1. Pulse Duration

The shock front of the laser-induced plasma and the cavitation bubble are potentially significant sources of mechanical damage to the surrounding tissue. The magnitude of the pressure amplitude and the size of the cavitation bubble scales with the laser pulse energy. In other words, the shorter the pulse duration, the less energy it transports at the same high intensity (Figure 1) [47]. Focusing a 100 fs pulse on a spot of only a few μm in diameter reduces the pulse energy for optical breakdown to a level of 1 μJ [48]. For this reason, thermal and mechanical effects of femtosecond pulses are significantly decreased and have only a very small impact.

Pulse duration, beam focusing, and wavelength can significantly influence the required pulse energy. Basically, it can be expected that the shorter the pulse duration, the less energy is required, and thus a better quality treatment of the tissue could be expected. However, for technical reasons the pulse length cannot be shortened arbitrarily without great investments. One important aspect to keep in mind is that the shorter the pulse, the broader the spectral band width. As a consequence, broadband laser sources would be required, which excludes many robust and economical laser designs. On the other hand, a pulsed laser beam disperses inside materials, which spreads the pulse apart, i.e., each wavelength component has its own speed of propagation. The shorter the pulse duration, the higher the pulse spread. In the beam delivery system of a surgical laser, one can compensate for this dispersion by an appropriate choices of glass types. At pulse durations around 10 fs, however, the short path of a laser pulse through the cornea and anterior chamber has to be taken into account. As a result, compensation can only be achieved at one depth and femtoseconds can become picoseconds at other depths. Considering that each eye differs slightly from the other, these effects are not easily compensated [49].

Another effect related to the pulse duration can also compromise cut quality. It has been demonstrated that with a pulse duration of less than 100 fs, other non-linear effects arise, such as self-focusing. The laser pulse induces a refractive index in the material that is smaller at the edges of the pulse than at its center. The material, therefore, acts like a lens on the laser pulse and focuses it towards its center. This effect is known as the optical Kerr effect and is also called self-focusing because it is caused by the laser pulse itself. This can cause spontaneous optical breakdown at an unwanted location, where streaks of chromatic aberrations are produced. Uncontrolled additional damage to the tissue can occur. Although such effects require certain combination of high pulse energies and low numerical aperture it is safe to say that a pulse duration of 150–200 fs, maximum up to 500 fs, seems to be the most suitable for microsurgical applications at the present time.

### 4.2. Laser Beam Size and Beam Focusing

The precision of laser surgery is directly related to the size of the laser focus. Note that the larger the focus diameter, the more energy the laser pulse must carry to reach the intensity threshold for photodisruption. The volume of laser focus is a function of the lateral focus diameter and its axial extent, the so-called Rayleigh range. Assuming a Gaussian beam profile, the focus volume V is given by the relationship

$$V=\frac{\lambda^{3}}{\pi^{3}}\times \frac{1}{{\mathrm{NA}}^{4}}$$

where λ is the wavelength and NA is the so-called numerical aperture of the focusing optics [50], which determines how small a laser beam can be focused. Noteworthy is the extremely strong dependence in the 4th power of the focus volume on the NA.

**Figure 2:**
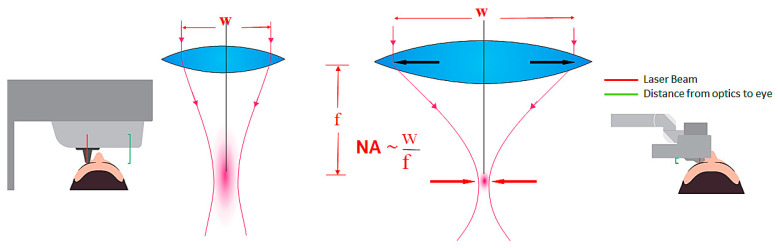
Middle: At a given working distance, increase of NA requires larger focusing optics. At large working distance (system to the left) the NA is limited to about 0.3. Substantial reduction of working distance (system to the right) allows for further increase of the numerical aperture, while shrinking the optics at the same time at the cost of scanning the focusing optics. The numerical aperture (NA) is defined as the quotient of W (optics diameter) and focal length (f).

Conventional laser systems in ophthalmology have a moderately high numerical aperture (NA = 0.2–0.3). There are two ways to create a high NA. One way is to increase the diameter of the optical system, which is technically very complex and demanding. A second way is to reduce the working distance which can also reduce the size of the optical system. To do this, one must bring the optics very close to the target object and, moreover, move them over the target area to get a correspondingly large lateral work area. Only the high-frequency femtosecond laser LDV (Ziemer) has gone this way providing substantially higher numerical apertures. Within a handpiece a movable microscope lens with a very short working distance scans across the eye. With this concept smallest pulse energies in the lower the nJ range (down to 10 nJ) are feasible. The larger the NA is, the smaller the cutting volume is, but also the necessary threshold energy. This also significantly reduces the probability of tissue damage due to cavitation bubbles and pressure waves (Figure 2).

**Figure 3:**
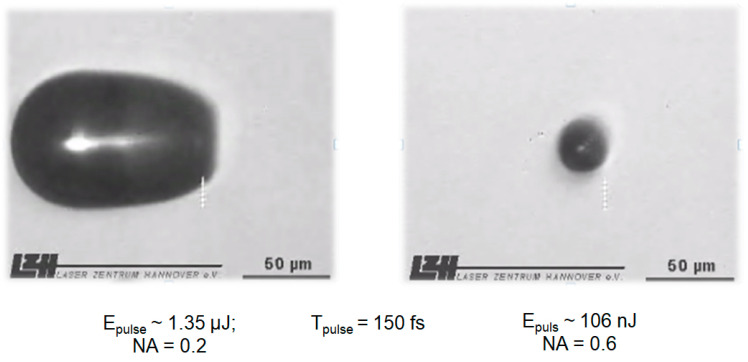
Cavitation in water for different of laser settings.

In this sense, with a smaller NA of, for example, 0.2 and an energy of ~1.35 µJ at 150 fs application time, the cavitation bubble is much larger than with an NA of 0.6 and an energy of 106 nJ at the same exposure time. With a larger cavitation bubble, the side effects are potentially increased [26,27,51] (Figure 3).

### 4.3. Femtosecond Laser Wavelength

Currently, all femtosecond laser systems in ophthalmology operate in the near-infrared range of 1030 to 1064 nm. The near-infrared laser light penetrates far enough into the ocular media without being scattered or absorbed. Furthermore, maximum permissible exposure to the eye using near-infrared is highest when compared to the visible spectral range.

There are two directions to shift the wavelength, one is to the infrared and the other to the UV range. A laser beam source at a wavelength of 1600–1650 nm has a better cut quality, especially for pathological corneas such as opacities or edema. These reduce scattering compared to lasers in the near-infrared wavelength range. It has been shown in vitro that femtosecond lasers in the 1600–1650 nm range need less energy and create less spot blurring than femtosecond lasers in the near-infrared range [52].

There are also proposals to apply shorter wavelengths in the UV spectral range. In the UV range, a photon carries a multiple of energy compared to the infrared photons. As a result, fewer photons are needed per pulse to cause photodisruption. This reduces the threshold energy and the associated collateral damage. In addition, the shorter wavelength can be more tightly focused. In this way, laser interventions could become even more precise. Furthermore, the cutting precision is determined by the focus volume, which in turn depends directly on the wavelength [49]. In a laboratory study, a UV femtosecond laser at a wavelength of 345 nm was able to reduce gas formation by a factor of 4.2 compared to an infrared femtosecond laser in the range of 1040 nm. However, it was also found that the UV laser source led to significantly more keratocyte death than the IR laser source [53]. As the threshold energy for photodisruption decreases with decreasing wavelength, UV photons allow longer laser pulses with higher pulse energy (µJ) to produce comparable precision in the tissue [54]. However, this circumstance also brings with it the danger of intensifying haze formation.

## 5. Currently Available Femtosecond Laser Devices

Currently, there are few lasers at or near the point of commercial release, including conventional femtosecond laser LensSx (Alcon Laboratories Inc., Fort Worth, TX, USA), Catalys (Abbott Medical Optics, Santa Ana, CA, USA), LensAR (LensARInc, Orlando, FL, USA), Victus (Technolas Perfect Vision and Bausch & Lomb, Rocherster, New York, NY, USA) and the high-frequency low-energy femtosecond laser Femto LDV Z8 (Ziemer Ophthalmic Systems AG, Port, Switzerland). All laser systems share a common platform which includes an anterior-segment imaging system, patient interface and femtosecond laser image to calculate and deliver the laser pulses.

## 6. Clinical Outcome

The use of the femtosecond laser offers a great advantage in the treatment of cataracts, especially in difficult cases. Especially in Marfan syndrome or traumatic cataracts with subluxated lens or intumescent and brunescent cataracts the use of femtosecond laser has great advantages. The intraoperative Optical Coherence Tomography (OCT) analysis of the anterior section structure makes a high application accuracy possible. This allows a so-called customized treatment.

The removal of a white intumescent cataract in the anterior bulbus section presents a challenge to surgeons. Intumescent cataracts often have increased intralenticular pressure because of the liquefaction of the cortex. Sometimes there remains a soft and liquid lentil nucleus, but there are also those with a brunescent hard lens nucleus. The most important, but also the most difficult step in a hypermatured white cataract is the capsulorhexis, especially in there is increased intralenticular pressure [55]. In manual capsulorhexis, one attempts to stabilize the anterior capsule by intracameral pressure using viscoelastic as a counterpressure so that it does not run out as soon as a cystotome has begun to form the capsulorhexis. One study of intumescent cataracts found incomplete capsulorhexis in 28% of eyes and posterior capsular tear in 1.9% [56]. In a regular cataract, incomplete capsulorhexis and capsule complications rates of 0.8–4.0% are reported [57,58,59]. Furthermore, in intumescent cataracts, trypan blue is added to stain the capsule making the capsulorhexis more visible. However, trypan blue is toxic to corneal endothelium, which can lead to a corneal decompensation. The femtosecond laser offers substantial advantages for intumescent cataract surgery. For instance, no trypan blue must be used. Moreover, the capsulotomy is cut very precisely, and one finds very good lens fragmentation in most cases. All these factors reduce capsule complication, EPT, and intraocular manipulation, thus resulting in less endothelial cell loss. In particular, the application of the femtosecond laser does not lead to an Argentine flag syndrome [60]. Various application variations have been described in the literature. An interesting surgical procedure is to initially cut a minicapsulotomy with a diameter of 2 mm by means of a femtosecond laser (CATALYS) to release the intracapsular pressure and then expand the capsulotomy to 5 mm in a second step [61]. With the minicapsulotomy, the likelihood of the capsule leaking out or the loss of the nucleus into the vitreous is significantly reduced.

Manual capsulorhexis can be challenging, especially in the cases of lens subluxations, trauma, or Marfan syndrome. Thanks to precise analysis by OCT, traumatic cataracts, especially anterior capsule laceration, can be performed safely using FLACS. Femtosecond laser is an asset in these high-risk cases, where capsulotomy can be customized in position and size [62]. Another case study described how FLACS can be used on a subluxated lens in the context of Marfan syndrome. Here, too, a sufficiently large, well-centered circular capsulotomy can be formed in the foreground that provides the greatest possible stability [63].

Intraoperative floppy iris syndrome (IFIS) is characterized by a floppy, atrophic iris that may prolapse during cataract surgery, resulting in progressive miosis. This has been associated with tamsulosin and other agents [64]. The potential for complications during cataract surgery in patients with IFIS is increased. Typically, iris trauma or posterior capsular rupture is described in the literature [65,66]. Several studies have shown that FLACS treatment is very safe in IFIS. A complete and strong capsulotomy reduces the likelihood of iris damage in intraoperative miosis and significantly reduces posterior capsular rupture [36,67,68,69].

During the technological development of the femtosecond laser with even lower energies, adaptive optics with customize, different energy levels depending on the tissue and depth of the planned application have emerged and there are further advantages in their application. The application speed is also getting faster and faster, resulting in shorter surgery times regardless of the system. In a recently published paper, in addition to a higher precision with regard to capsulotomy diameters, it was also shown that depending on the grade of the lens, the femtosecond laser results in significantly less endothelial cell loss [70]. Further clinical studies are needed to confirm these results. We are in the middle of a femtosecond laser development that still promises great potential for the future.

## 7. For the Future—One Laser for All Procedures

The outstanding feature of the femtosecond laser is its ability to cut tissue three-dimensionally throughout the anterior segment of the eye with utmost precision and minimal collateral damage. Because of the high space requirements of a femtosecond laser it makes sense to design a laser system for various surgical applications. The criteria for an “all-in-one” laser are high cutting quality in the area of all treated tissues, high stability of the laser source, and mobility (so that it can be used in various locations). The shift from the cornea to the lens, requires high-end focusing optics and the ability to change the pulse energies depending on the tissue structure.

For a corneal application, e.g., when cutting a corneal flap, very smooth cut surfaces need to be created to avoid additional scattering and aberrations after the treatment. The precision should be maintained over a comparatively large diameter, e.g., >12 mm. The shorter Rayleigh range and lower pulse energy are required for better precision. This is best achieved with high NA optics and low pulse energy [26,50]. The laser optics must be designed and manufactured in such a way to make the focus of the cornea as sharp as possible. This is achievable by an applanation of the cornea. In the deeper layers of the eye, e.g., in anterior chamber and the lens, the laser beam travels through curved surfaces and media with different refractive indices, which can cause significant aberrations of the laser beam. Consequently, laser application in deeper eye locations require laser sources and optics that are adapted for such applications. The technology of the LDV Z8 laser combines these features with adaptable pulse energies and optics. This leads to a very good cut quality at all tissue levels with correspondingly good clinical results.

## 8. Conclusions

The femtosecond laser technology is trend-setting especially in the clinical application in eye surgery. Through further development of the femtosecond laser, it will be possible to perform surgical procedures even more gently and quickly. On the other hand, the range of indications will be expanded in the future. Hopefully, in the future, conditions will be created where the introduction of the femtosecond laser will find its way into daily medical care, similar to the introduction of phacoemulsification.

## Data Availability

Not applicable.
